# Symptomatic venous thromboembolism associated with peripherally inserted central catheters predicts a worse survival in nasopharyngeal carcinoma: results of a large cohort, propensity score–matched analysis

**DOI:** 10.1186/s12885-018-5213-9

**Published:** 2018-12-29

**Authors:** Yu-Jing Liang, Lin-Quan Tang, Xue-Song Sun, Yu-Ying Fan, Jin-Jie Yan, Yu-Yun Du, Shan-Shan Guo, Li-Ting Liu, Hao-Jun Xie, Sai-Lan Liu, Qing-Nan Tang, Xiao-Yun Li, Hai-Qiang Mai, Qiu-Yan Chen

**Affiliations:** 1 Department of Nasopharyngeal Carcinoma, Sun Yat-sen University Cancer Center, 651 Dongfeng Road East, Guangzhou, 510060 People’s Republic of China; 2 State Key Laboratory of Oncology in South China, Collaborative Innovation Center for Cancer Medicine, Guangdong Key Laboratory of Nasopharyngeal Carcinoma Diagnosis and Therapy, Sun Yat-sen University Cancer Center, Guangzhou, 510060 China

**Keywords:** Symptomatic venous thromboembolism, Peripherally inserted central catheters, Nasopharyngeal carcinoma, Survival

## Abstract

**Background:**

Despite increasing use, symptomatic venous thromboembolism (VTE) associated with peripherally inserted central catheter (PICC) is a common complication in nonmetastatic nasopharyngeal carcinoma (NPC) patients.

**Methods:**

A total of 3012 nonmetastatic NPC patients were enrolled in this retrospective study, and we applied Cox regression and log-rank tests to assess the association between PICC-VTE and survival using the propensity score method (PSM) to adjust for gender, age, radiotherapy technique, tumor stage, node stage, UICC clinical stage and pre-treatment EBV DNA.

**Results:**

217 patients developed PICC-VTE, with an incidence of 7.20%. PSM identified 213 patients in the cohort with VTE and 852 in that without. Patients who developed PICC-VTE had a shorter 5-year PFS (77.5% vs 87.6%, *p* < 0.001), DMFS (85.0% vs 91.2%, *p* < 0.001), LRRFS (93.9% vs 97.7%, *p* < 0.001) and OS (85.4% vs 87.6%, *p* < 0.001). Subgroup analyses indicated that no significant survival difference was found between PICC-related superficial venous thrombosis and deep vein thrombosis, nor did different anticoagulant treatment methods.

**Conclusions:**

PICC-VTE was associated with a worse survival outcome in nonmetastatic NPC patients. A prospective randomized clinical trial is required to verify the results.

**Electronic supplementary material:**

The online version of this article (10.1186/s12885-018-5213-9) contains supplementary material, which is available to authorized users.

## Introduction

Nasopharyngeal carcinoma (NPC) is considered to be a rare form of cancer worldwide, accounting for 0.6% of all cancers. An estimated of 86,700 new cases of NPC have been reported, resulting in 50,800 deaths in 2012. Although NPC is uncommon globally, with a unique pattern of geographic and racial distribution, it is a leading cause of death in Asia, especially in a number of provinces in South-Eastern China like Guangdong, Hong Kong and Fujian [1, 2]. Radiotherapy is the cornerstone treatment modality and concurrent chemoradiotherapy with single-agent cisplatin regimen is recommended for locoregionally advanced NPC. In order to prevent renal impairment, intensive hydration is required before and during chemotherapy. In addition, parenteral nutrition support is urgently needed because of the difficulty in feeding caused by radioactive mucositis. Therefore, peripherally inserted central catheter (PICC) is frequently used in NPC patients nowadays, which makes intravenous injection, blood drawing and parenteral nutrition support much more convenient.

However, in spite of these strengths, PICC also contains a relatively high risk of catheter-associated venous thromboembolism (VTE), a quite common and potentially dangerous complication [3]. Furthermore, since intravenous infusion was administered for NPC patients for a long duration, they experience long term stay in bed or without active moving, which contributes to the formation of VTE. We previously reported that the incidence of PICC-VTE in NPC patients was 5.6% [4], and the figure tended to be even higher when asymptomatic thromboembolism was taken into consideration [5]. Although many cases of VTE are moderate and can be cured, it may cause recurrence, post-thrombotic syndrome or even pulmonary embolism [6, 7]. Plenty of researches have demonstrated that cancer patients with thromboembolism complications are associated with a lower survival rate [8, 9]. However, data of the prognosis of VTE induced by PICC in NPC patients are poorly documented. Therefore, we carried out a study to identify whether PICC-VTE has an influence on the prognosis of NPC patients.

## Patients and methods

### Patients population

**Fig. 1:**
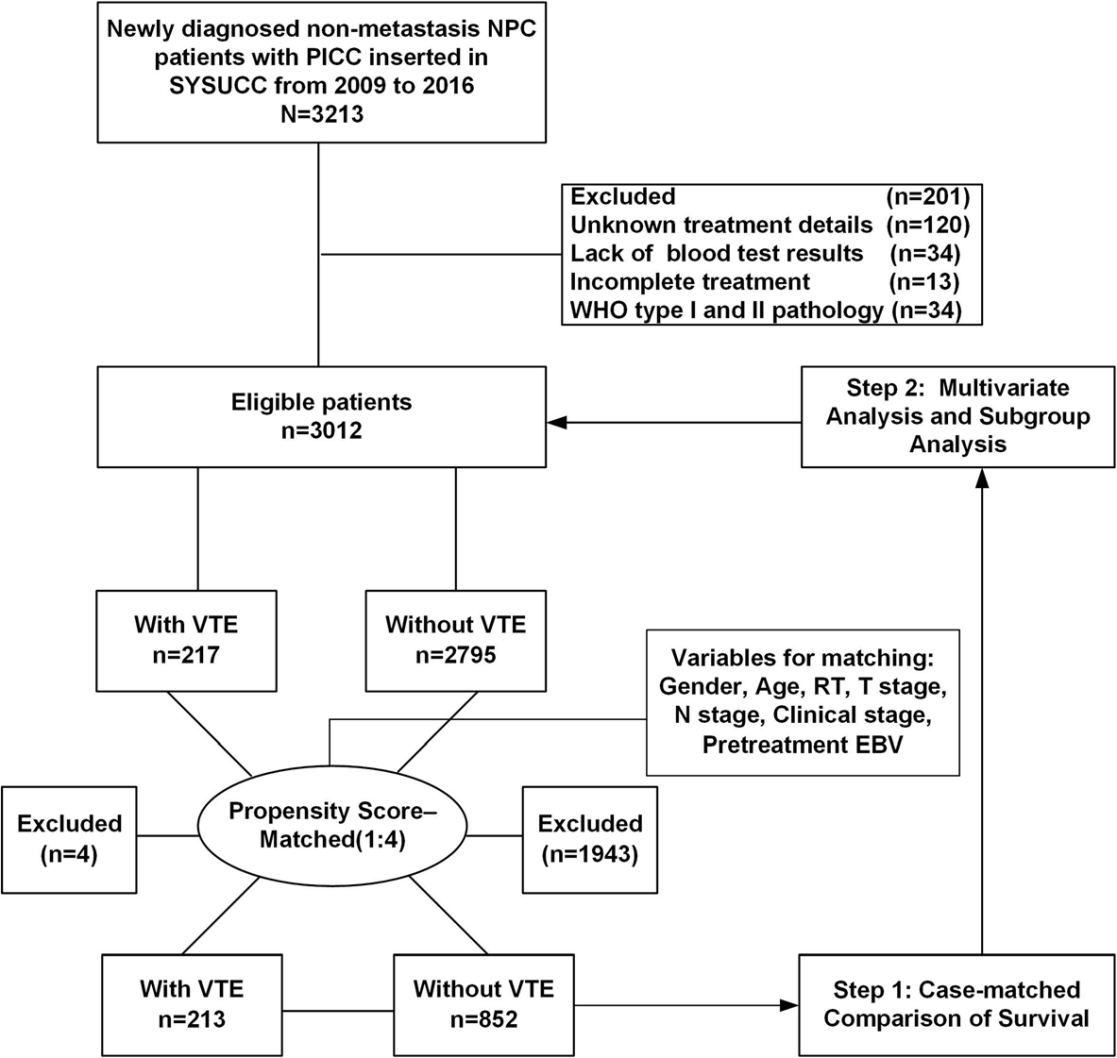
Analysis flow chart

In this retrospective cohort analysis, 3213 newly diagnosed non-metastasis NPC patients with PICC insertion between January 19, 2009 and September 30, 2016 were screened from an inpatient database on the computer in Sun Yat-sen University Cancer Center (SYSUCC). The disease was restaged according to the International Union Against Cancer/American Joint Committee on Cancer (UICC/AJCC) TNM classification (8th edition,2017). The pathology classification in our manuscript was the WHO 1991 pathology classification. The inclusion and exclusion criteria of our study was described in our flow chart (Fig. 1). Eventually 3012 patients were enrolled in the entire cohort. This study was approved by the clinical research ethics committee of SYSUCC and all patient provided written informed consents.

### Diagnosis and treatment

A sequence of evaluations was performed before treatment, including physical examination of head and neck, fibreoptic nasopharyngoscopy, magnetic resonance imaging (MRI) with contrast of head and neck, Positron emission tomography–computed tomography (PET/CT) or chest X-ray/ computed tomography (CT) with contrast, abdominal ultrasound/ CT with contrast and bone scan as alternative. Blood routine, biochemistry routine and EBV DNA were routinely measured before and during treatment. Dental examination is emphasized before radiotherapy. Different treatment methods were performed according to tumor extent and patients’ own situation.

Platinum-based concurrent chemoradiotherapy with or without neoadjuvant chemotherapy was recommended for patients with locoregional advanced NPC. Common neoadjuvant chemotherapy regimens included TPF: paclitaxel (135 mg/m^2^ d1) or docetaxel (60–75 mg/m^2^ d1) plus cisplatin (70 mg/m^2^ d1–3) plus 5-fluorouracil (3–3.75 g/m^2^, 120 h), PF: cisplatin (80/m^2^ d1) combined with 5-fluorouracil (4 g/m^2^, 120 h), GP: cisplatin (80 mg/m^2^ d1) with gemcitabine (1 g/m^2^ d1,8) and TP: docetaxel (75 mg/m^2^ d1) or paclitaxel (175 mg/m^2^ d1) plus cisplatin (80 mg/m^2^ d1).

Radiotherapy was conducted 5 times a week at 2 Gy/d by two-dimensional conventional radiotherapy (2D-CRT) or intensity modulated radiotherapy (IMRT). The accumulated radiation dosage of the primary tumor was 68~70 Gy. 3 courses of cisplatin were delivered at 80~100 mg/m^2^ during concurrent chemotherapy. And chemotherapy regimen was delivered every 3 weeks intravenously.

If symptoms indicate suspicious VTE, such as arm edema, swelling or pain, screening was performed and VTE was diagnosed through color Doppler ultrasound by an experienced sonographer. The diagnosis was made when a vein cannot be compressed since the thrombus prevented the vein from collapsing and blood flow [10]. Blood coagulation function was tested for patients with suspected VTE. Anticoagulants were not routinely used unless VTE developed.

### Follow-up and outcome

Patients were assessed every 3 months for the first 3 years and then every 6 months thereafter until death. Physical examination of head and neck, fibreoptic nasopharyngoscopy, magnetic resonance imaging (MRI) with contrast of head and neck, chest X-ray, abdominal ultrasound and blood tests were routinely performed annually during follow up. PET/CT and other examination would be considered by clinician if necessary. The primary endpoint in our study was progression-free survival (PFS), which was the length of time between the date of pathological diagnosis to the date of first failure or death from any cause. And our secondary endpoints were distant metastasis-free survival (DMFS), locoregional recurrence-free survival (LRRFS) and overall survival (OS). OS was defined as the time from the date of diagnosis to the date of death from any cause. LRRFS was defined as the time from the date of diagnosis to the date of first local and/or regional failure or death from any cause while that of DMFS was the time of distant metastasis or death from any cause.

### Statistical analysis

In our study, continuous variables were converted to categorical variables on the basic of routine cutoff points in clinical applications [11–14]. The continuous variables with asymmetric distribution were expressed as median (IQR), while symmetric distribution data were demonstrated as mean ± SD, and categorical variables were reported as the number of cases (%). Univariable and multivariable cox regression were used to estimate the hazard ratios (HRs) and 95% confidence intervals (CIs) for the correlations between variables and survival. Backward: conditional method was used in the multivariable cox regression. The propensity score for each patient was calculated to estimate their probability of VTE using multivariable logistic regression models given the following covariates: gender, age, radiotherapy technique, tumor stage, node stage, UICC clinical stage and pre-treatment EBV DNA. Matching was performed with the use of a 1:4 matching protocol without replacement (greedy-matching algorithm), with a caliper width equal to 0.01 of the standard deviation of the logit of the propensity score. Statistical analyses were conducted by Statistical Package for Social Sciences 24.0 (IBM Corporation, Armonk, NY, USA), R (http://www.R-project.org) and EmpowerStats software (www.empowerstats.com, X&Y solutions, Inc. Boston MA). All statistical tests were two-tailed. For a given variable, standardized differences of less than 10.0% suggest a relatively small imbalance and *P* < 0.05 was considered to indicate statistical significance.

## Results

### Patients’ characteristics

Propensity score matching (PSM) identified 213 patients in the cohort with VTE and 852 in that without. Baseline covariates in the PSM cohort were well-balanced and there was no statistically significant difference in covariates between 2 groups (Table 1). In our PSM cohort, the percentage of patients receiving neoadjuvant chemotherapy was 62.91% (670/1065), of which 71 patients developed PICC-VTE while that of concurrent chemoradiotherapy was 37.09% (395/1065) and 142 patients developed PICC-VTE.

**Table 1:** Descriptive characteristics of patients in the propensity score–matched cohort

Variables	Without VTE *n* = 852	With VTE *n* = 213	Standardized difference	*P* value
Age, yr	45.59 ± 11.54^#^	45.32 ± 10.88^a^	0.024	0.760
EBV DNA, copy/ml (%)			0.036	0.581
≤ 4000	438 (51.41%)	114 (53.52%)		
> 4000	414 (48.59%)	99 (46.48%)		
Gender (%)			0.038	0.686
Female	214 (25.1)	57 (26.8)		
Male	638 (74.9)	156 (73.2)		
Radiotherapy (%)			0.041	0.925
2DRT	5 (0.6)	2 (0.9)		
IMRT	847 (99.4)	211 (99.1)		
Tumor stage (%)				0.973
1	34 (4)	9 (4.2)	0.012	
2	97 (11.4)	24 (11.3)	0.004	
3	422 (49.5)	102 (47.9)	0.033	
4	299 (35.1)	78 (36.6)	0.032	
Node stage (%)				0.556
0	55 (6.5)	11 (5.2)	0.055	
1	253 (29.7)	67 (31.5)	0.038	
2	353 (41.4)	95 (44.6)	0.064	
3	191 (22.4)	40 (18.8)	0.090	
UICC clinical stage (%)				0.937
2	28 (3.3)	8 (3.8)	0.026	
3	378 (44.4)	95 (44.6)	0.005	
4	446 (52.3)	110 (51.6)	0.014	

Values were numbers (percentages) of patients unless stated otherwise

^a^ was reported as mean ± SD

Of the 3012 NPC patients enrolled in the entire cohort, 217 patients developed VTE, with an incidence of 7.20%. No patient developed recurrence, pulmonary embolism or life-threatening bleeding caused by VTE treatment during follow up. A majority of thrombosis were formed in more than one vein and 135 of them have thrombosis in at least one deep vein, accounting for 62.21%. The most common PICC associated VTE involved were basilic vein, subclavian vein and axillary vein. Features of PICC-VTE were listed in Table 2.

**Table 2:** Features of PICC-VTE in NPC patients

Variables	No. of patients (%)
Gender	
Female	58 (26.7)
Male	159 (73.3)
Age,yr	45.29 ± 11.13^b^
Insertion arm	
Dominant	135 (62.21)
Non-dominant	82 (37.79)
Insertion days	32.00 (59.00)^a^
Insertion vein	
Basilic	213 (98.16)
Cephalic	3 (1.38)
Median antebrachial	1 (0.46)
Thrombosis position	
Superficial vein	82 (37.79)
Deep vein involved	135 (62.21)
Pulmonary embolism	0 (0)
Treatment	
LMW heparin	35 (16.13)
Warfarin	25 (11.52)
Rivaroxaban	77 (35.48)
Others	80 (36.87)

*Abbreviations*: *LMW heparin* Low-molecular-weight heparin

Values were numbers (percentages) of patients unless stated otherwise

^a^was presented as median (IQR)

^b^ was reported as mean ± SD

Pre-treatment coagulation function between the VTE group and non-VTE group were compared in the entire cohort. Platelets, international normalized ratio and fibrinogen degradation product were significantly different. The value of International normalized ratio (INR) was lower in the VTE group while the values of platelets (Plt) and Fibrinogen degradation product (FDP) were significantly higher (Table 3).

**Table 3:** Comparison of pre-treatment coagulation tests in the entire cohort

	Without VTE *n* = 2795	With VTE *n* = 217	*P* value
Plt	238.12 ± 61.52	246.81 ± 64.12	0.046
PT	11.18 ± 0.80	11.11 ± 0.81	0.235
PT%	103.04 ± 17.26	105.52 ± 17.53	0.068
INR	0.99 ± 0.07	0.97 ± 0.07	< 0.001
APTT	25.96 ± 3.51	26.15 ± 2.90	0.471
FBG	3.00 (2.56–3.60)^a^	3.09 (2.62–3.41)^a^	0.780^c^
TT	17.96 ± 1.39	18.08 ± 1.53	0.291
DD	0.30 (0.17–0.60)^a^	0.27 (0.17–0.42)^a^	0.127^c^
FDP	1.00 (0.70–1.50)^a^	1.15 (0.70–2.00)^a^	0.020^c^

*Abbreviations*: *PLT* Platelets, *PT* Prothrombin time, *INR* International Normalized Ratio, *APTT* Activated Partial Thromboplastin Time, *FBG* Fibrinogen, *TT* Thrombin time, *DD* D-Dimer, *FDP* Fibrinogen Degradation Product

Values were reported as mean ± SD unless stated otherwise

^a^was presented as median (IQR)

^c^*P* values were calculated by Kruskal-Wallis test

### Survival analyses

**Fig. 2:**
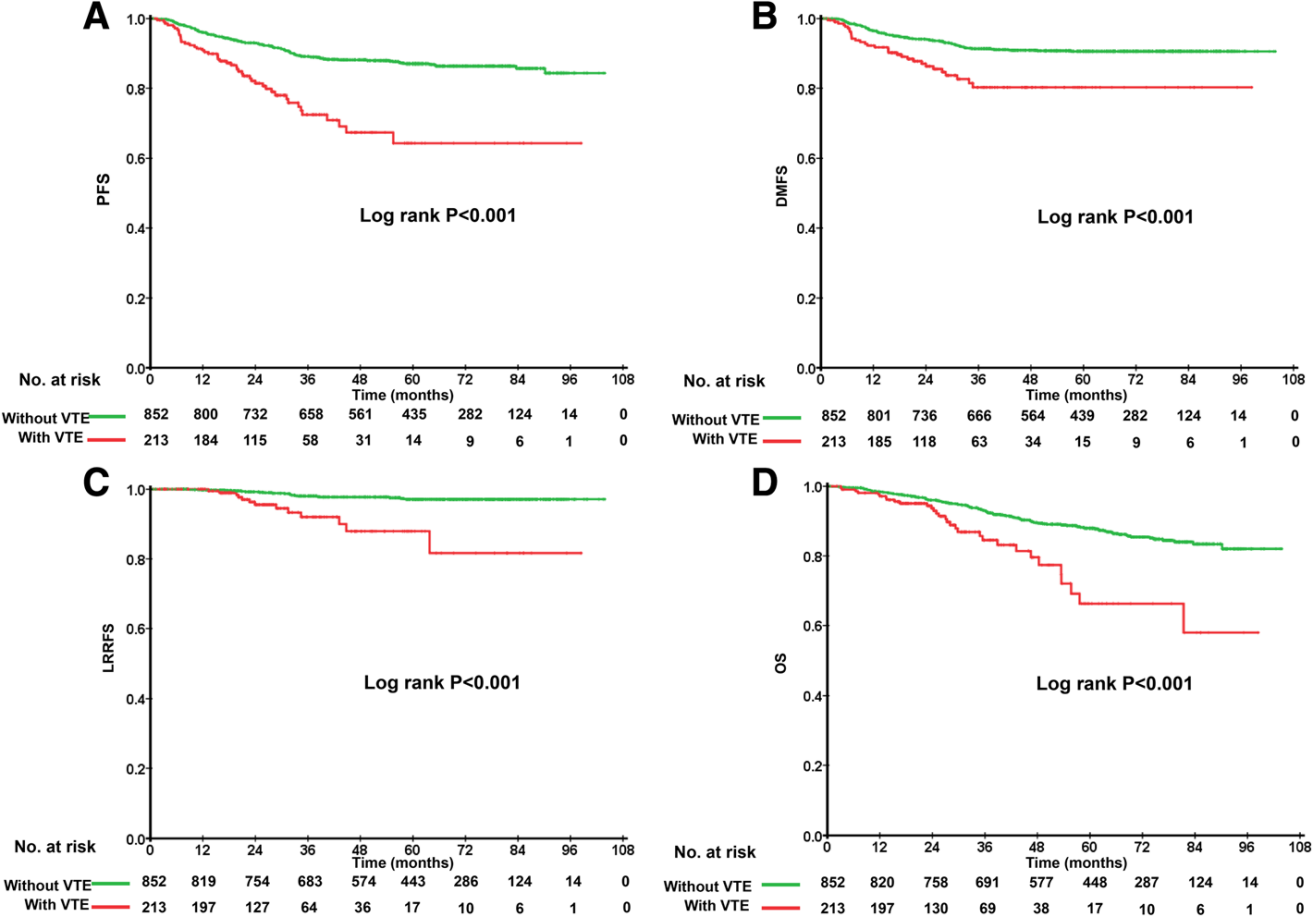
Kaplan-Meier curves of (**a**) progression free survival; (**b**) distant metastasis free survival; (**c**) locoregional recurrence-free survival; (**d**) overall survival with the VTE group and non-VTE group associated with PICC

The median follow-up time of the PSM cohort was 55.0 months (range = 0 to 102 months). During follow up, the number of patients who got local and/or regional recurrence was 13 versus (vs) 20, while 32 and 75 patients developed distant metastasis in the VTE and non-VTE group, respectively. The 5-year PFS, DMFS, LRRFS, and OS rates were 85.5, 90.0, 96.9 and 87.1%. For patients with and without PICC-VTE, the 5-year PFS, DMFS, LRRFS, and OS rates were 77.5% vs 87.6% (*p* < 0.001, Fig. 2a), 85.0% vs 91.2% (*p* < 0.001, Fig. 2b), 93.9% vs 97.7% (*p* < 0.001, Fig. 2c), 85.4% vs 87.6% (*p* < 0.001, Fig. 2d) respectively. In the univariable analysis, the hazard ratio (HR) for VTE was 2.79 (95% CI, 1.97–3.96, *p* < 0.001). After multivariable adjustments, VTE maintained a strong predictor with a HR of 2.92 (95% CI, 2.06–4.15, *p* < 0.001) (Table 4).

**Table 4:** Univariable and Multivariable Cox Proportional Hazards for prediction of progression in the well-balanced cohort

Predictors	Univariable analysis		Multivariable analysis	
	HR (95% CI)	*p* value	HR (95% CI)	*p* value
Gender				
Female	1.00 (ref.)	–	Not included	
Male	1.40 (0.94, 2.07)	0.095		
Age,yr				
≤ 45	1.00 (ref.)	–	1.00 (ref.)	–
> 45	1.42 (1.03, 1.96)	0.032	1.49 (1.07, 2.06)	0.017
ECOG scale				
0–1	1.00 (ref.)	–	Not included	
2	0.92 (0.13, 6.58)	0.935		
Smoking				
No	1.00 (ref.)	–	Not included	
Yes	1.18 (0.86, 1.64)	0.309		
NPC family history				
No	1.00 (ref.)	–	Not included	
Yes	0.96 (0.58, 1.59)	0.882		
Tumor stage				
1	1.00 (ref.)	–	1.00 (ref.)	–
2	1.61 (0.46, 5.64)	0.460	0.89 (0.24, 3.23)	0.856
3	1.65 (0.52, 5.25)	0.400	0.97 (0.29, 3.31)	0.966
4	3.32 (1.05, 10.52)	0.041	1.25 (0.36, 4.30)	0.729
Node stage				
0	1.00 (ref.)	–	Not included	
1	1.12 (0.54, 2.29)	0.764		
2	1.11 (0.55, 2.24)	0.767		
3	1.54 (0.75, 3.18)	0.240		
UICC clinical stage				
2	1.00 (ref.)	–	1.00 (ref.)	–
3	3.72 (0.51, 26.98)	0.194	3.66 (0.50, 26.65)	0.200
4	8.68 (1.21, 62.16)	0.032	7.56 (1.05, 54.55)	0.045
Radiotherapy				
2DRT	1.00 (ref.)	–	Not included	
IMRT	1.14 (0.16, 8.13)	0.898		
BMI, kg/m2				
< 18.5	1.00 (ref.)	–	1.00 (ref.)	–
18.5–23.9	0.63 (0.38, 1.03)	0.066	0.64 (0.38, 1.06)	0.080
24–27.9	0.68 (0.40, 1.15)	0.150	0.79 (0.46, 1.35)	0.384
≥ 28	0.31 (0.12, 0.77)	0.012	0.35 (0.14, 0.88)	0.025
EBVDNA, copy/mL				
≤ 4000	1.00 (ref.)	–	1.00 (ref.)	–
> 4000	2.27 (1.63, 3.16)	< 0.001	2.07 (1.47, 2.91)	< 0.001
HGB, g/L				
< 113	1.00 (ref.)	–	Not included	
113–151	3.09 (0.76, 12.52)	0.114		
≥ 151	2.70 (0.66, 11.08)	0.169		
hs-CRP, g/mL				
< 1.0	1.00 (ref.)	–	1.00 (ref.)	–
1.0–3.0	0.92 (0.60, 1.41)	0.689	0.76 (0.49, 1.17)	0.210
≥ 3.0	1.53 (1.03, 2.27)	0.036	1.20 (0.80, 1.80)	0.375
LDH, U/L				
< 245	1.00 (ref.)	–	Not included	
≥ 245	1.58 (0.97, 2.59)	0.066		
PICC-VTE				
No	1.00 (ref.)	–	1.00 (ref.)	–
Yes	2.79 (1.97, 3.96)	< 0.001	2.92 (2.06, 4.15)	< 0.001

*Abbreviations*: *2DRT* Two-dimensional radiotherapy, *EBV* Epstein-Barr virus, *ECOG* Eastern Cooperative Oncology Group, *hs-CRP* high-sensitivity C-reactive protein, *IMRT* Intensity-modulated radiotherapy, *LDH* Serum lactate dehydrogenase levels, *HGB* Hemoglobin, *NPC* Nasopharyngeal carcinoma, *UICC* Union for International Cancer Control, *BMI* Body Mass Index, *PICC* Peripherally Inserted Central Catheter, *VTE* Venous thromboembolism, *ref*. reference, *CI* Confidence intervals

### Subgroup analysis

**Fig. 3:**
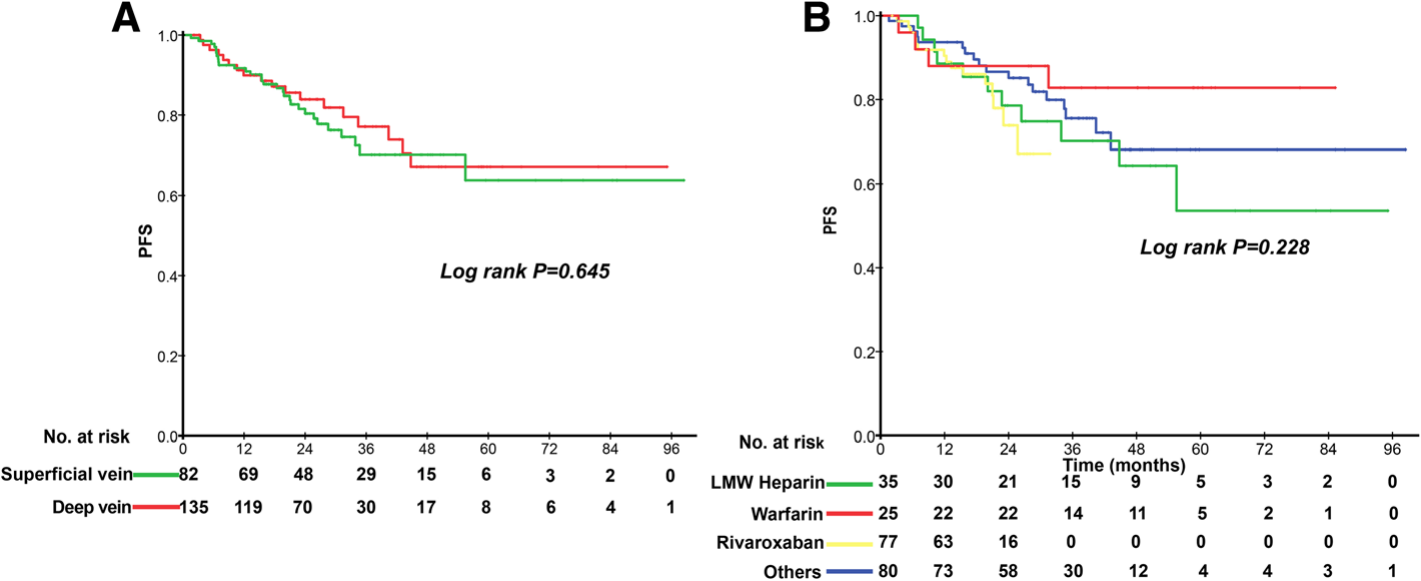
Kaplan-Meier curves for progression free survival according to (**a**) PICC-related superficial venous thrombosis and deep vein involved thrombosis; (**b**) different anticoagulant treatment methods of patients who developed VTE in the entire cohort

We are interest that whether there is a survival difference between PICC-related superficial venous thrombosis and deep vein involved thrombosis in NPC patients. Moreover, Low molecular weight (LMW) heparin and warfarin had been the most commonly used anticoagulants. And the ideal anticoagulant Rivaroxaban that appeared in recent years was also widely used. Therefore, we also conducted a subgroup analysis to further investigate the survival of different treatment methods of PICC-VTE in NPC patients. With regard to the patients with superficial PICC induced venous thrombosis and deep vein involved thrombosis, no significant differences in 5-year PFS were found (*P* = 0.645, Fig. 3a). Nor did different treatment methods have significant impact on 3-year PFS (*P* = 0.228, Fig. 3b).

### Sensitivity analysis

In a multivariable cox regression analysis of PICC-VTE versus non PICC-VTE for prediction of survival in the matching cohort, PICC-VTE was associated with significantly higher rates of progression (HR = 2.92, *p* < 0.001), distant metastasis (HR = 2.34, *p* < 0.001), locoregional recurrence (HR =4.79, *p* < 0.001) and death (HR = 2.47 *p* < 0.001). In a multivariable cox regression analysis of the entire cohort, similar risks of survival outcomes were maintained (Additional file 1: Table S1).

## Discussion

To the best of our knowledge, this is the first nasopharyngeal cancer-specific series to document that PICC-VTE correlate with poorer survival. PSM was performed to balance the characteristics between the VTE group and non-VTE group.

VTE is a common complication in cancer patients and is regarded as a leading cause of death. The incidence of VTE in NPC patients in our analysis was 7.20%, which was in accordance with studies with previous documented of 1~20% [5]. VTE has been reported to be a significant predictor of decreased survival in several malignant diseases, including lung, gastrointestinal, prostate and breast cancer, with HRs ranging from 1.9 to 5 [15, 16]. Chew et al. conducted a large cohort of 235,149 cases s to investigate the incidence of VTE and its effect on survival among patients with common cancers including prostate, breast, lung, melanoma, non-Hodgkin lymphoma, gastrointestinal and urogenital cancer. VTE maintained a significant predictor of decreased survival in all cancer types in the first year with adjustment for age, race, and stage of cancer in the multivariate analysis (HRs, 1.6–4.2; *P* < 0.01, [17]). M Mandala and colleagues investigated the survival of 227 irresectable pancreatic cancer patients and compared PFS and OS between these patients with and without VTE during chemotherapy. Patients with occurrence of VTE during chemotherapy were reported to have significantly worse PFS and OS compared to patients without, with HRs of 3.04 and 1.95. At multivariate assessment with adjustment for age, tumor stage and chemotherapy, VTE during chemotherapy continued to be a significant predictor of decreased PFS with a 2.62-fold HR [18].

Consistently, in our study, developing symptomatic VTE associated with PICC was a prognostic parameter for shorter survival and led to increased risk of progression. With a median follow-up of 5 years, the HR of PFS in NPC patients with development of PICC-VTE in the well-balanced cohort was 2.92-fold (*p* < 0.001) higher than patients without. The 5-year PFS rate of NPC patients who developed PICC-VTE was 77.5%, which was significantly lower than that of patients without (87.6%, *P* < 0.001). Similar results were observed in the secondary endpoints, with 5-year DMFS, LRRFS, and OS rates of 85.0% vs 91.2% (*p* < 0.001), 93.9% vs 97.7% (*p* < 0.001), 85.4% vs 87.6% (*p* < 0.001) in the VTE group and non-VTE group respectively.

In our study, age, tumor stage, UICC clinical stage, BMI, EBV DNA, CRP and PICC-VTE were found to be associated with PFS in univariable analysis. After adjustment, multivariate analysis revealed that age, BMI, EBV DNA, UICC clinical stage and PICC-VTE were still significant independent factors for PFS in nonmetastatic NPC patients. Older patients had poorer progression free survival, which could theoretically be interpreted by more comorbidities and decreased physical function. The American Joint Committee on Cancer (AJCC) TNM classification is the most widely used cancer staging system and aims to provide reference of treatment regimens and prognosis in clinical. BMI and EBV DNA had been demonstrated to be correlated with survival in NPC patients [19].

Moreover, we discovered that PICC-VTE was a significant risk factor for survival, which has not been studied in NPC patients before. This effect was independent of age, EBV DNA level, UICC clinical staging and BMI. Taken together, we could completely confirm that PICC-VTE was an independent risk factor for survival in nonmetastatic NPC patients.

No significant survival difference was found between the PICC-related superficial venous thrombosis and deep vein involved thrombosis in NPC patients. Additionally, in spite of the lack of direct evidence on catheter-related thrombosis in cancer patients, the fundamental of anticoagulation therapy is to relieve acute symptoms, maintain PICC function and prevent post-thrombotic syndrome or even pulmonary embolism. Common treatment modalities include removal of catheter and anticoagulation [20, 21]. In our study, different treatment methods have no significant impact on survival in NPC patients. With regard to VTE, risk factors associated were patient-, provider-, and device-related characteristics. General items include stage of carcinoma, thrombosis history, age, comorbidities, catheter insertion, chemotherapy, surgery and others [22–24]. We previously have reported that VTE history, BMI, ECOG performance score and metastasis stage were significantly associated with symptomatic PICC-VTE in NPC patients [4]. In our study, the values of pre-treatment Plt and FDP were significantly higher in the VTE group. In addition, we also discovered that NPC patients developing VTE induced by PICC had a higher probability of progression and mortality rate. There are a few biological explanations that support our results. Previous studied have reported: (i) that NPC interacts with both the hemostatic system and coagulation system; (ii) that VTE is caused by prothrombotic factors produced by the malignancies, which can be exaggerated by cancer treatment modalities and exists persistently; (iii) that mechanisms involved in cancer associated hypercoagulability can lead to tumor progression and MP-enriched prothrombotic and proangiogenic factors play vital roles in supporting tumor growth [18, 25–27].

However, this study is not devoid of limitations. First, this retrospective study is suspected of selection bias, although we tried to eliminated selection biases by performing a propensity score matching analysis. Next, the incidence and influence of asymptomatic VTE were not taken into consideration in our study.

## Conclusion

In summary, symptomatic VTE associated with PICC is a common complication in nonmetastatic NPC patients, which predicts a worse survival outcome. No significant survival difference was found between PICC-related superficial venous thrombosis and deep vein involved thrombosis, nor did different anticoagulant treatment methods. A well-designed, multi-center, prospective, randomized study is needed to verify the results in the future.

## Additional files

Additional file 1: **Table S1.** PICC-VTE vs non PICC-VTE: Risk of primary and secondary outcomes in the PSM cohort and the entire cohort. (DOCX 19 kb)

## Abbreviations

2DRT: Two-dimensional radiotherapy
AJCC/UICC: American Joint Committee on Cancer//Union for International Cancer Control
APTT: Activated Partial Thromboplastin Time
BMI: Body Mass Index
CI: Confidence intervals
DD: D-Dimer
EBV: Epstein-Barr virus
ECOG: Eastern Cooperative Oncology Group
FBG: Fibrinogen
FDP: Fibrinogen Degradation Product
HGB: Hemoglobin
hs-CRP: High-sensitivity C-reactive protein
IMRT: Intensity-modulated radiotherapy
INR: International Normalized Ratio
LDH: Serum lactate dehydrogenase levels
NPC: Nasopharyngeal carcinoma
PICC: Peripherally Inserted Central Catheter
PLT: Platelets
PT: Prothrombin time
ref.: Reference
TT: Thrombin time
VTE: Venous thromboembolism
