# Supplementary material for: Symptomatic venous thromboembolism associated with peripherally inserted central catheters predicts a worse survival in nasopharyngeal carcinoma: results of a large cohort, propensity score–matched analysis
Source: BMC Cancer. 2018 Dec 29;18:1297. doi: 10.1186/s12885-018-5213-9 (PMC6310996; doi:10.1186/s12885-018-5213-9)
Supplement: Supplementary file 1 — Table S1. PICC-VTE vs non PICC-VTE: Risk of primary and secondary outcomes in the PSM cohort and the entire cohort. (DOCX 19 kb) [file 12885_2018_5213_MOESM1_ESM.docx]

Table S1 PICC-VTE vs non PICC-VTE: Risk of primary and secondary outcomes in the PSM cohort and the entire cohort

| Survival outcomes | No. of  Patients | No. of Patients with Events | Event Rate | Hazard Ratio  (95% CI) | P value |
| --- | --- | --- | --- | --- | --- |
| In the PSM cohort |  |  |  |  |  |
| Progression |  |  |  |  | p<0.001 |
| Non PICC-VTE | 852 | 106 | 12.44% | Ref |  |
| PICC-VTE | 213 | 48 | 22.54% | 2.92 (2.06 - 4.15) |  |
| Distant metastasis |  |  |  |  | p<0.001 |
| Non PICC-VTE | 852 | 75 | 8.80% | Ref |  |
| PICC-VTE | 213 | 32 | 15.02% | 2.34 (1.54 - 3.56) |  |
| Locoregional recurrence |  |  |  |  | p<0.001 |
| Non PICC-VTE | 852 | 20 | 2.35% | Ref |  |
| PICC-VTE | 213 | 13 | 6.10 % | 4.79 (2.32 - 9.87) |  |
| Death |  |  |  |  | p<0.001 |
| Non PICC-VTE | 852 | 106 | 12.44% | Ref |  |
| PICC-VTE | 213 | 31 | 14.55% | 2.47 (1.64- 3.74) |  |
| In the entire cohort |  |  |  |  |  |
| Progression |  |  |  |  | p<0.001 |
| Non PICC-VTE | 2795 | 310 | 11.09% | Ref |  |
| PICC-VTE | 217 | 48 | 22.12% | 2.58. (1.89-3.52) |  |
| Distant metastasis |  |  |  |  | p<0.001 |
| Non PICC-VTE | 2795 | 217 | 7.76% | Ref |  |
| PICC-VTE | 217 | 32 | 14.75% | 2.22. (1.52-3.24) |  |
| Locoregional recurrence |  |  |  |  | p<0.001 |
| Non PICC-VTE | 2795 | 66 | 2.36% | Ref |  |
| PICC-VTE | 217 | 13 | 5.99% | 3.62 (1.98-6.64) |  |
| Death |  |  |  |  | p<0.001 |
| Non PICC-VTE | 2795 | 306 | 10.95% | Ref |  |
| PICC-VTE | 217 | 31 | 14.29% | 2.18 (1.50-3.18) |  |

Abbreviations: PICC = Peripherally Inserted Central Catheter; VTE = venous thromboembolism; ref = reference; CI= confidence intervals
